# The American Journal of Science and Arts

**Published:** 1850-12

**Authors:** 


Art. XIII.—*The American Journal of Science and Arts.* Conducted by Professors B. Silliman, and B. Silliman, jr., and James D. Dana. Second series. No. 30. November 1850. With an Index to Vols. I—X.

This Journal, alike creditable to science, and to American erudition, has just reached us in its 30th number. This number is filled with an unusual variety of scientific papers of great interest. For the benefit of such of the readers of the *Western Journal*, as are fond of scientific subjects, not directly connected with medicine, we present a copy of the table of contents of the November number of the Journal of Science:

Address of Sir David Brewster before the twentieth meeting of the British Association at Edinburgh, July 31, 1850.

On the proper height of lightning rods; by Prof. Elias Loomis.

On the electrical phenomena of certain houses; by Prof. Elias Loomis.

On a new method of decomposing Silicates in the process of analysis; by Henry Wurts.

On the availability of the Green sand of New Jersey as a source of Potash and its compounds; by Henry Wurts.

On the Diurnal and Annual variations in the declination of the Magnetic Needle, and in the horizontal and vertical magnetic intensities; by Prof. W. A. Norton.

On the analogy between the mode of reproduction in Plants, and the "alternation of generations" observed in some radiata; by James D. Dana.

On Electro-Magnetism as a moving power; by Prof. Chas. G. Page, M.D.

Singular property, and extraordinary size and length of the secondary spark; by Prof. Chas. G. Page, M.D.

On Rutile and Chlorite in Quartz; by Professor O. P. Hubbard, M.D.

Occurrence of chrystalized oxyd and chromium in furnaces for the manufacture of Chromate of Potash; by W. P. Blake.

Memoir on Emery; by J. Lawrence Smith, M.D. First part—On the Geology and Mineralogy of Emery, from observations made in Asia Minor.

On American Spodumene; by Geo. J. Brush.

Optical examination of several American micas; by B. Silliman, jr., A.M., M.D., &c.

Analysis of Phlogopite from St. Lawrence county, N. Y ; by Wm. J. Craw.

These are the titles of the leading papers, and there is no one of them that does not possess interest for the lover of natural science. The paper of Professor Silliman on several American micas is one of the best memoirs that we have seen, even from that fine mineralogist.

In addition to the contents we have named, there is a variety of miscellaneous intelligence of an instructive character, in this number. Its quality is good, but its quantity, compared with that of preceding numbers, is encroached upon by the general Index to the preceding 10 volumes of the Journal. This Index is of great value to those who are fortunate enough to possess the volumes of the Journal of Science.

The American reader will peruse with interest the remarks of Sir David Brewster, before the British Association for the advancement of science. The eulogium he bestows upon the discovery made by the humble American schoolmaster, of Pottsville, Mr. Kirkwood, is nothing more than what is due to that remarkable man. The appreciation of his merits, by such a philosopher as Sir David Brewster, must be highly satisfactory to Mr. Kirkwood and his numerous friends. Pottsville is a strange place to give birth to such a remarkable astronomical discovery as that announced by Mr. Kirkwood. We should sooner have supposed that Pottsville would come to want coal for fuel, than that such an out of the way place would have produced a man, who has lighted up a point in astronomy that was darkness to all the powerful and acute intellects that have been reading the heavens for ages.

We commend the Journal of Science to our readers. There is not an American reader of science whose heart does not glow with pride, in the consciousness that in Silliman's Journal, as the work is familiarly called, America possesses a scientific periodical fully equal in reputation to any in the world.

